# Conversion Surgery for Patients with Advanced Gastric Cancer with Peritoneal Carcinomatosis

**DOI:** 10.1155/2021/5459432

**Published:** 2021-11-10

**Authors:** Ting-Ying Lee, Guo-Shiou Liao, Hsiu-Lung Fan, Chung-Bao Hsieh, Teng-Wei Chen, De-Chuan Chan

**Affiliations:** Division of General Surgery, Department of Surgery, Tri-Service General Hospital, National Defense Medical Center, No. 325, Section 2 Cheng-Kong Rd., Neihu District, Taipei 114, Taiwan

## Abstract

**Background:**

Patients with advanced gastric cancer (AGC) with peritoneal carcinomatosis (PC) usually have poor outcomes and high mortality risk, even with cytoreductive surgery (CRS) and hyperthermic intraperitoneal chemotherapy (HIPEC). This study analyzed the prognostic factors of AGC with PC and evaluated laparoscopic HIPEC (LHIPEC) plus neoadjuvant intraperitoneal and systemic chemotherapy (NIPS) as a conversion surgery for AGC patients with PC with a poor initial prognosis. *Patient and Methods*. We retrospectively evaluated 127 patients with AGC and PC from January 1, 2012, to March 1, 2020. After the exclusion of 32 ineligible patients, the conversion group comprised 34 patients who underwent LHIPEC + NIPS as a conversion surgery followed by CRS plus HIPEC. The CRS + HIPEC group included 15 patients who underwent CRS with HIPEC alone. Additionally, the C/T group comprised 23 patients who received systemic chemotherapy, and the palliative group comprised 23 patients who received only conservative therapy or palliative gastrectomy.

**Results:**

The conversion group demonstrated a significantly better mean overall survival compared to the CRS + HIPEC, C/T, and palliative groups (*p* < 0.001). Patients in the conversion group who underwent LHIPEC + NIPS had significantly decreased peritoneal cancer index (PCI) scores (*p* < 0.001) and ascites (*p*=0.003). Malignant ascites amount also significantly decreased after treatment in the LHIPEC + NIPS group (*p* < 0.001).

**Conclusions:**

LHIPEC + NIPS can significantly improve the overall survival, the PCI score, and malignant ascites amount in peritoneal cytology-positive gastric cancer with PC, and an initially high PCI score. Therefore, it may be a feasible conversion strategy for AGC patients with PC.

## 1. Introduction

Gastric cancer has an incidence of 8.2% in cancer patients worldwide; it is the third most common cancer and remains a global healthcare problem [1]. Although recent advances and screening efforts have resulted in earlier detection in most endemic areas, the majority of patients are diagnosed at an advanced stage, which is traditionally considered the terminal stage of the disease because most of these patients expire within three months without treatment [2]. Systemic chemotherapy is prescribed for the treatment of advanced gastric cancer (AGC) with peritoneal carcinomatosis (PC) in the National Comprehensive Cancer Network guidelines for gastric cancer [3, 4]. However, even among patients undergoing systemic chemotherapy, the majority die within one year [2, 5]. The treatment strategy for AGC with PC is progressing slowly.

In the early 1990s, PC was still considered the terminal stage of gastric cancer [6]. Recently, the use of multimodality treatment, including cytoreductive surgery (CRS), combined with hyperthermic intraperitoneal chemotherapy (HIPEC), has led to promising results in selected AGC patients with PC [7].

However, the prognosis of AGC with PC after CRS combined with HIPEC is still variable [8, 9]. Some studies have even advocated that HIPEC is less beneficial in patients with PC from gastric cancer than in patients with PC from other malignancies [10, 11]. In the presented literature, positive ascites cytology, high peritoneal cancer index (PCI) scores (PCI > 12), and inadequate complete cytoreduction (CC-2 or CC-3) have been reported as poor prognostic factors of CRS + HIPEC [11–15]. Therefore, the challenges for improving the prognosis of AGC with PC post CRS + HIPEC are in treating and eliminating the positive ascites cytology, diminishing the PCI score, and eradicating the intra-abdominal cancer cell during operation [15, 16].

In some studies, 70% of patients with PC showed a PCI score above 12 and positive peritoneal cytology at the time of diagnosis. Moreover, complete cytoreduction could only be performed in 30% of the patients at the time of diagnosis [17, 18]. The aims of conversion therapy are (i) reduction of the PCI and (ii) eradication of peritoneal cavity micrometastases and peritoneal free cancer cells. The first study to consider the use of HIPEC as a neoadjuvant approach prior to gastrectomy in patients with a positive peritoneal cytology or low-volume PC was published in 2017 by Badgwell et al. In this single-arm phase II trial, 19 patients received laparoscopic HIPEC [19]. Approximately half of the patients (48%) underwent two to five laparoscopic procedures. After the final HIPEC, seven patients had negative peritoneal cytology and no PC, and five of these patients underwent definitive surgery.

In 2006, a new bidirectional chemotherapeutic strategy for patients with PC from gastric cancer was proposed by Yonemura et al., which included neoadjuvant intraperitoneal and systemic chemotherapy (NIPS) [20]. In this study, patients who had undergone a minimum of two cycles and up to six cycles of NIPS were considered prior to the cytoreduction. Treatment resulted in negative peritoneal cytology in 56% of the patients, and those who underwent a complete resection had a median survival of 20.4 months compared to 14.4 months in all patients. Similar to the 2012 study by Yonemura et al., complete response was achieved in 24% and complete cytoreduction was achieved in 68% of the patients following neoadjuvant laparoscopic HIPEC (LHIPEC)+NIPS [15]. Notably, 78 patients with ascites also showed an improvement in their symptoms in this study, further establishing the role of NIPS as a palliative technique.

The aim of this study was to evaluate LHIPEC combined with NIPS as a conversion surgery for AGC patients with PC complicated by an initially high PCI and sympatric ascites.

## 2. Materials and Methods

### 2.1. Patients

**Figure 1:**
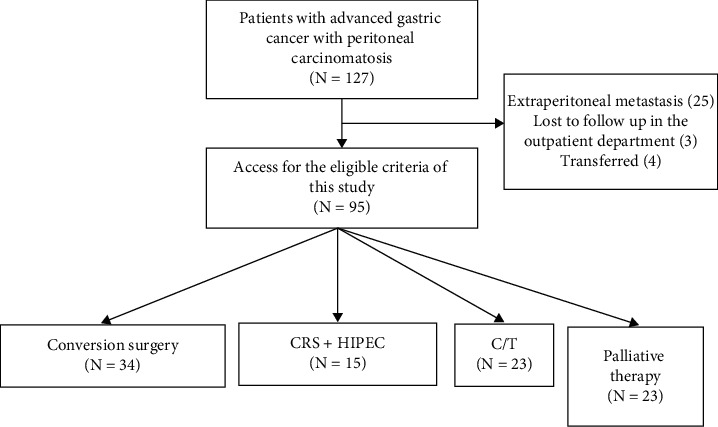
Flowchart of the study participants. CRS + HIPEC: cytoreductive surgery + hyperthermic intraperitoneal chemotherapy; C/T: chemotherapy.

We retrospectively reviewed 127 AGC patients diagnosed with PC at the Tri-Service Medical Center between January 1, 2012, and December 1, 2019. Extensive diagnostic workup was performed in all cases with thoracic-abdominopelvic computed tomography (CT) with double contrast; positron emission tomography was selectively performed in doubtful cases. We excluded patients with extra-peritoneal metastasis (*N* = 25), patients lost to follow-up at the outpatient department (*N* = 3), and those transferred to another hospital (*N* = 4) (Figure 1). Eventually, 95 cases were enrolled and separated into four groups: (1) conversion surgery group: these patients underwent LHIPEC + NIPS and received CRS + HIPEC (*N* = 34), (2) CRS + HIPEC group: these patients initially received CRS + HIPEC (*n* = 15), (3) C/T group: patients only received systemic chemotherapy (*N* = 23), and (4) palliative therapy group: patients who underwent palliative gastrectomy or only received hospice care (*N* = 23). This study was conducted in accordance with the Declaration of Helsinki, received *a priori* approval from our Institutional Ethics Committee, and was registered with the Institutional Review Board of the Tri-Service General Hospital (TSGH-IRB No: B202105039). Written informed consent was obtained from the patients for publication of this cohort study and any accompanying images.

We evaluated the clinical characteristics and OS of the four groups. The perioperative factors of CRS + HIPEC, including operation time, blood loss, visceral resection, complete cytoreduction, and complications in the conversion surgery group and CRS + HIPEC group were also evaluated. We also assessed the amount of ascites, peritoneal positive cytology conversion rate, PCI score, and patient performance before and after LHIPEC + NIPS therapy in the conversion surgery group. The ascitic amount and PC score were evaluated and recorded at diagnostic laparoscopy each time. The peritoneal cytology data was also collected in each diagnostic laparoscopy and evaluated by conventional microscopic viewing and immunoassay.

The chemotherapy group received systemic chemotherapy with XELOX regimens first (14 patients, twelve 2-week cycles of intravenous capecitabine, 1,000 mg/m^2^ plus intravenous oxaliplatin 130 mg/m^2^ on day 1). The patients would change to the other regimens (DHFL + CDDP) until disease progression or intolerable complication was noted.

### 2.2. CRS + HIPEC

The main goal of CRS was to remove all visible disease by resecting the primary tumor by gastrectomy and lymphadenectomy and all peritoneal implants by peritonectomy and visceral resection. The aim of CRS was to obtain complete macroscopic cytoreduction as a precondition for the administration of HIPEC. Residual disease was classified intraoperatively using the completeness of cytoreduction (CC) score. [21] CC-0 indicates no visible residual tumor, CC-1 indicates residual tumor nodules ≤2.5 mm, CC-2 indicates residual tumor nodules between 2.5 mm and 25 mm, and CC-3 indicates residual tumor nodules *>*25 mm or a confluence of unresectable tumor nodules at any site within the abdomen and pelvis. CC-2 and CC-3 cytoreductions were regarded as incomplete. HIPEC was administered after CRS; all patients received a closed technique.

### 2.3. Treatment Protocol in the Conversion Surgery Group (Figure 2)

The conversion surgery group patients initially underwent a staging laparoscopy; ascites was suctioned and the volume was measured. Cytologic studies were conducted using the ascitic fluid or peritoneal washing fluid, and biopsy specimens were routinely taken from the peritoneal nodules. PC in the entire abdominal cavity was quantitatively evaluated using PCI. The small bowel and its mesentery were divided into four sectors (upper jejunum, lower jejunum, upper ileum, and lower ileum). Small bowel PCI (SB-PCI) was the sum of the lesion size scores for the four sectors. Following PCI determination, two longitudinal 5 cm incisions were made bilaterally in the lower abdomen. Four drainage tubes were placed (two in the bilateral subdiaphragmatic space for use as inlet tubes, one in the pelvic cavity, and one in the lower midline of the peritoneal cavity for use as an outlet tube). LHIPEC (oxaliplatin 400 mg or mitomycin-C 30 mg + paclitaxel 120 mg in 2,500 mL of 5% dextrose in saline for 90 min at 42°C) was performed if the PCI score was >12 or the SB-PCI score was >3. After LHIPEC was completed, a peritoneal port system (Hickman subcutaneous port; Bard Access Systems, Inc., Salt Lake City, UT, USA, 7.5Fr) was introduced into the abdominal cavity, and the tip was placed in the left upper quadrant adjacent to the stomach. Subsequently, we prescribed NIPS (systemic regimen: XELOX capecitabine 2,000 mg/m^2^ and oxaliplatin 130 mg/m^2^; intraperitoneal chemotherapy: paclitaxel 20 mg in 1000 mL of normal saline through the peritoneal port) per month for three months. After the first cycle, the patients underwent abdominal contrast CT and repeat staging laparoscopy. At the time of the second-staging laparoscopy, ascitic volume, peritoneal cytologic status, and PCI were again determined. If the PCI score was less than 12 and SB-PCI < 3, we prescribed cytoreductive surgery (CRS) plus HIPEC for the patient. However, if the PCI score continued to be more than 12 or SB-PCI > 3, LHIPEC was performed and NIPS treatment was administered for 3 months. After the second cycle of therapy was completed (three months later), the patient underwent repeat abdominal contrast CT and staging laparoscopy. The patients then underwent CRS plus HIPEC.

### 2.4. Statistical Analysis

Data on patient demographics, preoperative indicators of disease severity, comorbidity, patient performance based on the Eastern Cooperative Oncology Group (ECOG) and visual analog scale (VAS) scores, disease symptom variables, and mean follow-up duration were collected. The clinical characteristics were compared with the Chi-square test. The OS was compared with the Kaplan–Meier method. The prognostic factors were analyzed by univariate and multivariate analyses. Pre- and postintervention variables including the PCI score and amount of ascites were also recorded and compared with the *t*-test. Data management and statistical analyses were conducted using the SPSS statistical software (version 22.0; IBM, Chicago, IL, USA). A statistically significant value was defined as *p* ≤ 0.05.

## 3. Results

The clinical characteristics of the patients are shown in Table 1. There were no significant differences in age, sex, comorbidities (except for diabetes: *p*=0.044), and patient performance (ECOG) between the four groups. The VAS score showed the worst performance in the palliative group (*p*=0.028). With regard to the tumor stage, the CRS + HIPEC group demonstrated a lower T2 stage (*p*=0.001) and N1 stage (*p*=0.007). On evaluating the symptoms of the patients, there was no significant difference in epigastric pain, upper gastrointestinal (UGI) bleeding, and body weight loss among the four groups, which was expected to result in a higher frequency of ascites in the conversion surgery group (*p*=0.004) and higher frequency of gastric outlet obstruction in the CRS + HIPEC group (*p*=0.013). On laboratory examination, there was no significant difference in the white blood cell count, serum hemoglobin, and biochemistry, except for a higher creatinine level in the chemotherapy group (1.07 ± 0.65) and palliative group (1.07 ± 0.64, *p*=0.021).

**Figure 3:**
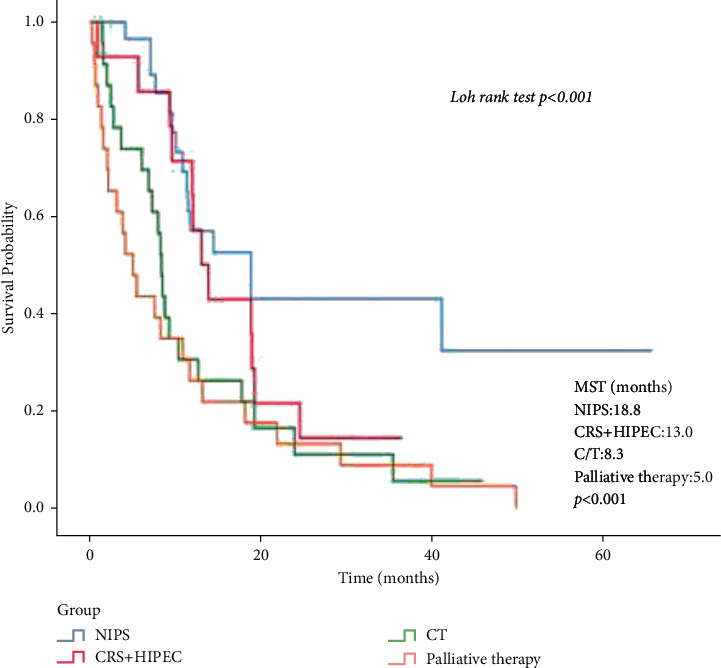
OS of the 95 gastric cancer patients with PC who underwent conversion surgery, CRS + HIPEC, systemic chemotherapy, and palliative therapy. NIPS: neoadjuvant intraperitoneal and systemic chemotherapy; CRS + HIPEC: cytoreductive surgery + hyperthermic intraperitoneal chemotherapy; C/T: chemotherapy; MST: median survival time (months).

The median survival time was significantly longer in the conversion group compared to the CRS + HIPEC, chemotherapy, and palliative groups (18.8 months, 13 months, 8.3 months, and 5 months, respectively; *p* < 0.001). Similarly, the OS was higher in the conversion group than in the other three groups (*p* < 0.001) (Figure 3).

The evaluation of perioperative factors of CRS + HIPEC is shown in Table 2. The initial PCI score was significantly higher in the conversion group (17.44 vs. 7.46, *p*=0.002). There was no difference in the number of visceral resections, CC, operation time, blood loss, and 1-year surgical mortality rate between the conversion and CRS + HIPEC groups. The surgical complication rate was 29.4% in the conversion group and 26.7% in the CRS + HIPEC group (*p*=0.894). The most common surgical complication in both groups was anastomotic leakage (15.2% vs. 14.3%, *p*=0.996).

On performing a prognostic analysis (Table 3), T and N stage, comorbidities, and symptoms including gastric outlet obstruction and UGI bleeding were not significantly associated with the 1-year OS. AGC patients with PC who were >65 years of age, had a PCI score ≥12, ascites, incomplete cytoreduction (CC-2), and higher ECOG (>3) had a significantly higher mortality risk. There was also a higher mortality rate if surgical complications were present.

**Figure 4:**
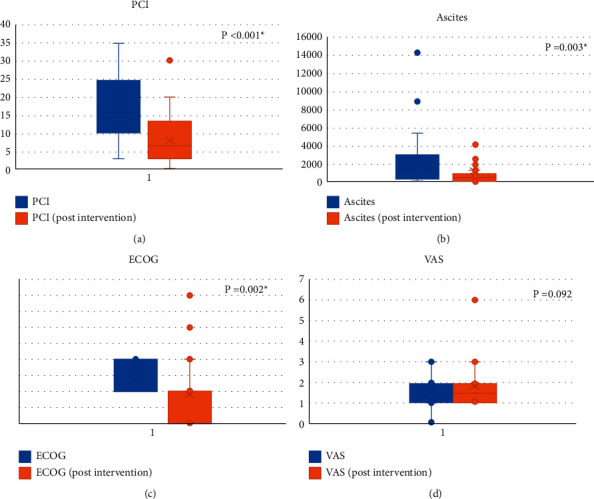
LHIPEC + NIPS intervention in the conversion group patients. LHIPEC: laparoscopic hyperthermic intraperitoneal chemotherapy; NIPS: neoadjuvant intraperitoneal and systemic chemotherapy; PCI: peritoneal cancer index; ECOG: Eastern Cooperative Oncology Group; VAS: visual analog scale. (a) PCI. (b). Ascites. (c) ECOG. (d) VAS.

In the conversion surgery subgroup analysis (Table 4 and Figure 4), the PCI score (17.44 ± 8.9 vs. 8.83 ± 6.1, *p* < 0.001) and amount of ascites (1974.12 ± 829.6 ml vs. 442.65 ± 22.6 ml, *p*=0.003) showed a significant decrease after the LHIPEC + NIPS intervention. Patient performance (ECOG) also improved significantly after the LHIPEC + NIPS intervention (0.441 ± 0.01 vs. 0.882 ± 0.12, *p*=0.002). There was no significant difference in the VAS scores between preintervention and postintervention LHIPEC + NIPS patients (*p*=0.092). Malignant ascites also showed a significant decrease after intervention (55.8% ⟶ 8.2%, *p* < 0.001).

## 4. Discussion

In our study, patients with AGC and PC showed poor outcomes, even when they were treated with various systemic chemotherapy regimens. Most of the patients survived for more than 1 year. This is consistent with other studies [5, 22]. Few selected patients who underwent CRS + HIPEC demonstrated significantly improved survival times. All our patients had PC (M1 stage). The majority of palliative group patients showed the image stage without surgical intervention. We believe that there was some bias in this variable. The univariate analysis also showed that the T and N stages were not the prognostic factors in AGC with PC. We believe that the advanced T (*p*=0.001) or N (*p*=0.007) stage in the palliative group did not influence the outcome. The prognostic factors included age, ECOG, ascites, PCI score, CC score, and surgical complications of AGC with PC. Kitayama et al. reported that when PCI > 12, CRS + HIPEC has less benefit in AGC with PC, even completeness of cytoreduction (CC-0) [8]. Malignant ascites also had a 40% recurrence rate with curative surgery [23, 24]. Therefore, the major challenges for improving the survival rate of CRS + HIPEC are the PCI score and malignant ascites. Conventional systemic chemotherapy possesses minimal advantages for PC because of the existence of a blood-peritoneal barrier [25]. Intraperitoneal (IP) chemotherapy offers potential therapeutic advantages over systemic chemotherapy by generating high local concentrations of chemotherapeutic drugs in the peritoneal cavity. This advantage of IP chemotherapy is evident in the area under the curve ratio of intraperitoneal versus plasma exposure [15, 26]. In the conversion surgery group, the preintervention PCI score, ascites amount, and positive cytology conversion rate were significantly better than the preintervention values. This is concurrent with the findings of Canbay et al. who first described the use of neoadjuvant intraperitoneal chemotherapy combined with systemic chemotherapy in a large single-center case series in Japan with 194 patients [27]. Among these, 152 (78%) patients whose cytology became negative were classified as responders. Yonemura et al. also demonstrated in 52 patients that the use of neoadjuvant LHIPEC + NIPS can significantly improve the PCI score compared to LHIPEC alone [28]. Complete cytoreduction (CC-0) was also achieved in 57.6% of the patients in the LHIPEC + NIPS group. Furthermore, the OS also improved due to the improved PCI score and control of ascites.

Surprisingly, the ratio of complete cytoreduction (CC0-1) and surgical complication rate was not associated with an added benefit for the conversion group as compared to the CRS + HIPEC group in this study. The PCI score was significantly improved in the conversion group after LHIPEC + NIPS therapy. Although we thought that the initial PCI score would be higher in the conversion group (mean PCI: 17.4), following conversion therapy, the PCI score was equal between the two groups. The rate of surgical complications in CRS + HIPEC has been shown to be 5% to 40% [29, 30]. The most common surgical complication is anastomotic leakage, and this may lead to significant mortality even after curative cytoreduction (CC0). Previously, immature surgical techniques and aggressive eradication of small bowel implants resulted in major complications of anastomotic leakage and intra-abdominal abscess in our patients. In this study, we avoided eliminating the serosal implants and tumor nodules near the small bowel mesentery. This significantly reduced the postoperative leakage rate.

According to the results of our study, LHIPEC + NIPS may not improve the PCI score or ascites amount. Nevertheless, it can significantly convert the positive cytology of ascites to negative. The continually high intraperitoneal chemotherapeutic concentration can eradicate peritoneal cavity micrometastases and peritoneal free cancer cells. This may significantly improve the OS. In this study, we did not use the standard questionnaire to measure the patients' quality of life. Instead, we used the ECOG patient performance score which also significantly improved after the LHIPEC + NIPS intervention, and there was no obvious unfavorable effect on VAS. This may be attributed to the control of the malignant ascites complications including ileus, refractory peritonitis, poor appetite, and partial bowel obstruction by LHIPEC + NIPS [13, 28]. The symptoms were significantly relieved, and this improvement might have improved the quality of life. Most patients tolerated this therapy and experienced minimal side effects. Some of the patients in the conversion surgery group who underwent NIPS showed a significant improvement in the ascites-related complications (data not shown). They also demonstrated a significantly improved quality of life after the initial treatment and refused further surgical intervention (CRS + HIPEC). However, the serum tumor markers became elevated and the disease relapsed after 6 months of NIPS treatment in these patients. Testing for cytotoxic drug resistance was performed even after we shifted to another regimen. Although these patients finally received CRS + HIPEC, gastric cancer recurred and progressed within 3 months. Therefore, when the disease symptoms and PCI scores have been successfully controlled after 6 months, primary tumor resection and CRS + HIPEC should be suggested as soon as possible. Even if the PCI score was not controlled successfully (PCI score persistently > 12) after NIPS treatment for 6 months, we believe that CRS + HIPEC is indicated because drug resistance may increase.

The limitations of our study are its small size and its retrospective and single-center design. Patient group selection may cause selection bias. Some of our patients underwent palliative gastrectomy in the palliative and C/T groups. The OS did not benefit from palliative gastrectomy due to the small case number. Further, postoperative care and adjuvant chemotherapy could not be completely analyzed. Moreover, the follow-up time should be longer. A larger prospectively randomized clinical trial comparing CRS + HIPEC followed by LHIPEC + NIPS and CRS + HIPEC alone is necessary to assess the clinical benefits of this treatment strategy.

## 5. Conclusions

In conclusion, LHIPEC + NIPS is a feasible and reasonable treatment strategy as a conversion surgery for peritoneal cytology-positive gastric cancer with PC and an initially high PCI score. However, further prospective trials comparing LHIPEC + NIPS and neoadjuvant systemic chemotherapy are necessary.

## Figures and Tables

**Figure 2:**
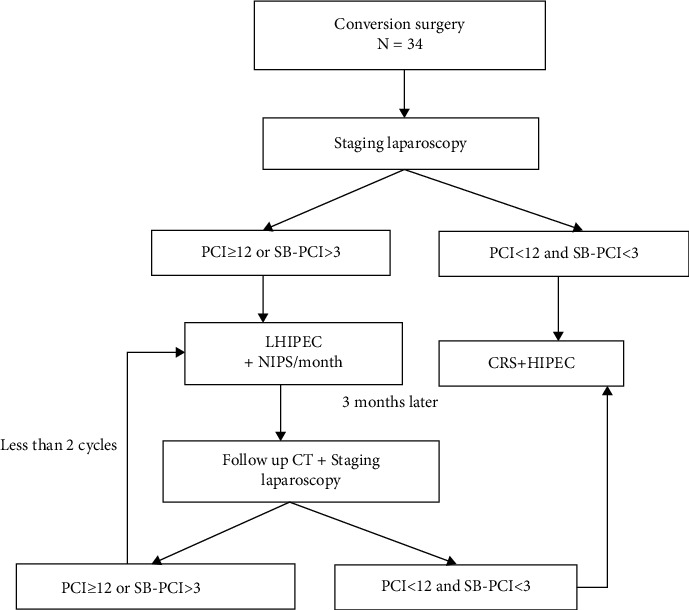
A schematic diagram of the treatment protocol in the conversion group. PCI: peritoneal cancer index; SB-PCI: small bowel-peritoneal cancer index; CT: computed tomography; HIPEC: hyperthermic intraperitoneal chemotherapy; LHIPEC: laparoscopic HIPEC; NIPS: neoadjuvant intraperitoneal and systemic chemotherapy; CRS: cytoreductive surgery.

**Table 1:** Clinical characteristics of the patients.

Variable		NIPS	CRS + HIPEC	C/T	Palliative	*p*
		(*n* = 34)	(*n* = 15)	(*n* = 23)	(*n* = 23)	

Age (y/o)		65.3 (6.02)	66.07 (10.19)	65.91 (11.11)	67.43 (9.05)	0.850
Sex (male)		17 (51.52%)	8 (57.14%)	16 (69.57%)	15 (65.22%)	0.536
Comorbidity	HTN	7 (21.21%)	6 (42.86%)	10 (43.48%)	12 (52.17%)	0.095
	CAD	3 (9.09%)	3 (21.43%)	5 (21.74%)	8 (34.78%)	0.132
	Diabetes	1 (3.03%)	3 (21.43%)	6 (26.09%)	5 (21.74%)	0.044^*∗*^
	CVA	1 (3.03%)	1 (7.14%)	2 (8.7%)	1 (4.35%)	0.751
	ESRD	0 (0%)	0 (0%)	1 (4.35%)	3 (13.04%)	0.077
	Pulmonary	2 (6.06%)	1 (7.14%)	2 (8.7%)	1 (4.35%)	0.999
(PS) ECOG	0 (%)	10 (30.3%)	4 (28.57%)	11 (47.83%)	10 (43.48%)	0.425
	1∼2 (%)	21 (63.64%)	9 (64.29%)	12 (52.17%)	10 (43.48%)	
	>3 (%)	2 (6.06%)	1 (7.14%)	0 (0%)	3 (13.04%)	
VAS		1.48 ± 0.97	1.21 ± 0.43	1.7 ± 1.11	2.09 ± 1.38	0.028^*∗*^
TNM stage	T2 (%)	2 (6.06%)	0 (0%)	3 (13.04%)	6 (26.09%)	0.001^*∗*^
	T3 (%)	9 (27.27%)	1 (7.14%)	12 (52.17%)	10 (43.48%)	
	T4 (%)	22 (66.67%)	13 (92.86%)	8 (34.78%)	7 (30.43%)	
	N1 (%)	3 (9.09%)	1 (7.14%)	6 (26.09%)	7 (30.43%)	0.007^*∗*^
	N2 (%)	17 (51.52%)	6 (42.86%)	3 (13.04%)	7 (30.43%)	
	N3 (%)	7 (21.21%)	7 (50%)	11 (47.83%)	9 (39.13%)	
Symptoms	Ascites (%)	20 (60.61%)	2 (14.29%)	6 (26.09%)	6 (26.09%)	0.004^*∗*^
	Epigastric pain (%)	33 (100%)	13 (92.86%)	22 (95.65%)	20 (86.96%)	0.128
	UGI bleeding (%)	11 (33.33%)	4 (28.57%)	7 (30.43%)	10 (43.48%)	0.746
	Gastric outlet obstruction (%)	7 (21.21%)	10 (71.43%)	8 (34.78%)	8 (34.78%)	0.013^*∗*^
	BW loss (%)	18 (54.55%)	3 (21.43%)	13 (56.52%)	15 (65.22%)	0.068
Laboratory test	WBC (m/mm^3^)	7080.91 ± 2516.55	7208.57 ± 3637.68	8513.48 ± 2849.05	9082.61 ± 4607.45	0.117
	Hgb (g/dL)	10.94 ± 2.87	10.37 ± 2.66	10.95 ± 2.76	10.22 ± 2.74	0.728
	Sodium (mEq/L)	137.12 ± 3.62	137.43 ± 3.11	135.57 ± 3.92	137.13 ± 2.44	0.260
	Potassium (mmol/L)	3.67 ± 0.42	3.94 ± 0.41	3.82 ± 0.45	3.87 ± 0.48	0.193
	AST (U/L)	19.42 ± 9.86	21.64 ± 10.3	41.74 ± 75.95	56.7 ± 94.08	0.154
	Cr (mg/dL)	0.75 ± 0.22	0.88 ± 0.18	1.07 ± 0.65	1.07 ± 0.64	0.021^*∗*^
	Albumin (g/dL)	3.38 ± 0.56	3.46 ± 0.36	3.24 ± 0.48	3.06 ± 0.64	0.083

HTN, hypertension; CAD, coronary artery disease; CVA, cerebrovascular accident; ESRD, end-stage renal disease; PS (ECOG): patient performance (Eastern Cooperative Oncology Group); VAS: visual analog scale; UGI bleeding: upper gastrointestinal bleeding; BW loss: bodyweight loss; WBC: white cell count; AST: aspartate transaminase; Cr: creatinine; NIPS: neoadjuvant intraperitoneal and systemic chemotherapy; CRS + HIPEC: cytoreductive surgery + hyperthermic intraperitoneal chemotherapy; C/T: chemotherapy; Hgb: hemoglobin; ^*∗*^*p*<0.05.

**Table 2:** Surgical and postoperative characteristics in the conversion group and CRS + HIPEC group.

Variable		Conversion surgery	CRS + HIPEC	*p*
		(*n* = 34)	(*n* = 15)	

PCI score		17.44 ± 8.9	7.46 ± 5.3	0.002^*∗*^
Mean visceral resection (range)		2.87 (1–4)	2.28 (1–4)	0.882
Completeness of cytoreduction	CC-0 (%)	26 (76.7)	11 (73.3)	0.794
	CC-1 (%)	7 (20.4)	3 (25.0)	
	CC-2 (%)	1 (2.9)	1 (6.7)	
Operation time (min)		472.3 ± 165.3	490.3 ± 54.2	0.391
Blood loss (ml)		228.3 ± 108.8	285.7 ± 105.5	0.168
Complications	Bleeding (%)	1 (3.0)	1 (7.1)	0.556
	Anastomotic leakage (%)	5 (15.2)	2 (14.3)	0.996
	Infection (%)	4 (12.1)	1 (7.1)	0.802
	Total (%)	10 (29.4)	4 (26.7)	0.894
	1-year surgical mortality	80.0% (8/10)	75% (3/4)	0.982

CRS + HIPEC: cytoreductive surgery + hyperthermic intraperitoneal chemotherapy; CC: complete cytoreduction score; PCI: peritoneal cancer index.

**Table 3:** Univariate and multivariate analysis of prognostic factors in GC patients with PC.

Variables	Patient numbers	1-year OS	Median OS (months)	*p* value	Adjusted OR (95% CI)	*p* value

Gender				0.732		
Male	58	46.5%	18.8			
Female	37	48.6%	14.6			
Age				**<0.001^∗^**	3.8 (1.1–12.8)	**0.01^∗^**
≤65	54	55.7%	17.9			
>65	41	26.8%	8.3			
Stage				0.600		
T2	12	41.6%	11.4			
T3	32	43.7%	17.7			
T4	51	50.7%	15.3			
Lymph node status				0.690		
N1	28	39.1%	10.3			
N2	33	39.6%	14.6			
N3	34	42.2%	12.6			
PCI				**0.019^∗^**	3.5 (1.3–10.6)	**0.03^∗^**
<12	33	63.6%	14.6			
>12	16	26.9%	9.2			
Ascites				**0.032^∗^**	2.8 (1.1–8.8)	**0.02^∗^**
Yes	44	24.0%	7.9			
No	51	61.4%	19.2			
Complete of cytoreduction				**0.036^∗^**	2.6 (1.8–6.6)	**0.04^∗^**
CC-0	22	63.6%	18.8			
CC-1	11	27.2%	10.0			
CC-2	6	16.6%	8.3			
Comorbidity				0.420		
0	58	46.6%	16.4			
1–2	32	39.4%	9.6			
≧3	5	40.0%	10.8			
ECOG				**0.017^∗^**	2.16 (1.16–2.87)	**0.043^∗^**
0	47	52.3%	14.4			
1–2	33	36.3%	10.3			
≧3	15	15%	4.1			
UGI bleeding				0.359		
Positive	32	37.5%	13.9			
Negative	63	41.6%	14.6			
Gastric outlet obstruction				0.139		
Positive	31	35.1%	11.4			
Negative	64	37.7%	12.6			
Surgical complications				0.001^*∗*^	5.8 (3.9–21.2)	**0.001^∗^**
Yes	14	21.4%	7.0			
No	35	88.5%	20.7			

GC: gastric cancer; PC: peritoneal carcinomatosis; OS: OR: odds ratio; PCI: peritoneal cancer index; CC: complete cytoreduction score; ECOG: Eastern Cooperative Oncology Group patient performance score; UGI: upper gastrointestinal.

**Table 4:** Subgroup analysis of the LHIPEC + NIPS intervention in the conversion group patients.

LHIPEC + NIPS in the conversion group		Preintervention	Postintervention	*p*

PCI score		17.44 ± 8.9	8.83 ± 6.1	<0.001^*∗*^
Ascites	Amount (ml)	1974.12 ± 829.6	442.65 ± 22.6	0.003^*∗*^
	Positive cytology (%)	19 (55.4%)	3 (8.82%)	<0.001^*∗*^
PS (ECOG)		0.882 ± 0.12	0.441 ± 0.01	0.002^*∗*^
VAS		1.471 ± 0.47	1.882 ± 0.38	0.092

LHIPEC: laparoscopic hyperthermic intraperitoneal chemotherapy; NIPS: neoadjuvant intraperitoneal and systemic chemotherapy; PCI: peritoneal cancer index; PS (ECOG): patient performance (Eastern Cooperative Oncology Group); VAS: visual analog scale.

## Data Availability

All data generated or analyzed during this study are included in this published article.
